# Out-of-pocket expenditures for pharmaceuticals: lessons from the Austrian household budget survey

**DOI:** 10.1007/s10198-016-0797-y

**Published:** 2016-04-30

**Authors:** Alice Sanwald, Engelbert Theurl

**Affiliations:** Department of Economics and Statistics, University of Innsbruck, Universitätsstrasse 15, 6020 Innsbruck, Austria

**Keywords:** Out-of-pocket pharmaceutical expenditures, Consumer survey, Two-part model, Generalized linear model, Hurdle model, Zero-inflated negative binomial model, I1

## Abstract

**Background:**

Paying pharmaceuticals out of pocket is an important source of financing pharmaceutical consumption. Only limited empirical knowledge is available on the determinants of these expenditures.

**Objectives:**

In this article we analyze which characteristics of private households influence out-of-pocket pharmaceutical expenditure (OOPPE) in Austria.

**Design and methods:**

We use cross-sectional information on OOPPE and household characteristics provided by the Austrian household budget survey 2009/10. We split pharmaceutical expenditures into the two components prescription fees and over-the-counter (OTC) expenditures. To adjust for the specific characteristics of the data, we compare different econometric approaches: a two-part model, hurdle model, generalized linear model and zero-inflated negative binomial regression model.

**Findings:**

The finally selected econometric approaches give a quite consistent picture. The probability of expenditures of both types is strongly influenced by the household structure. It increases with age, doctoral visits and the presence of a female householder. The education level and income only increase the probability of OTC pharmaceuticals. The level of OTC expenditures remains widely unexplained while the household structure and age influence the expenditures for prescription fees. Insurance characteristics of private households, either private or public, play a minor role in explaining the expenditure levels in all specifications. This refers to a homogeneous and comprehensive provision of pharmaceuticals in the public part of the Austrian health care system.

**Conclusions:**

The article gives useful insights into the determinants of pharmaceutical expenditures of private households and supplements the previous research that focuses on the individual level.

## Introduction

Comparisons between OECD member states reveal that the out-of-pocket share of total pharmaceutical spending (41 % in 2011) is more than twice as much as the out-of-pocket share of total spending on health services (18 % in 2011) [1]. The empirical knowledge on the determinants of out-of-pocket pharmaceutical expenditures (OOPPE) is limited. One reason for this fact is the lack of adequate routine data on pharmaceutical expenditures on the individual and household level. To understand the possible covariates driving OOPPE and to select a sound econometric identification strategy require close insights into the interaction between the relevant actors in the decision-making process on pharmaceutical consumption. Such analyses end in preferred specifications of indicators for pharmaceutical use/expenditures and of possible influential covariates. Available routine data sets on pharmaceutical consumption provided by socioeconomic (e.g., SOEP in Germany) and health-related surveys (NHANES in the US, EHES in selected EU countries, ATHIS in Austria) fulfill such claims only to a limited extent; specific information on OOPPE is missing. In the following article we study the determinants of OOPPE in Austria using cross-sectional information from the latest national household budget survey conducted in 2009/10 [2]. We give insights into the socioeconomic determinants of OOPPE, an undertaking that is new for pharmaceutical spending in Austria. The article benefits from the voluminous previous research work on out-of-pocket health-care expenditures based on microdata in general [3–18] and on the scanty literature on OOPPE and respectively on self-medication [19–25]. In the following we briefly discuss the previous research work on OOPPE and self-medication. In a two-part model, Leibowitz et al. [21] study cost-sharing effects of prescription drug expenditures using individual panel data from the Rand Health Insurance Experiment. They find that drug expenditures respond to the cost sharing faced by the consumers. They also observe very significant site, age and sex effects. Based on a cross-sectional data set on an individual basis from Spain, Figueiras et al. [23] identify sociodemographic factors associated with self-medication (use of non-description drugs) using multivariate Cox’s regression. They find that self-medication is more prevalent among women, singles, persons living in large agglomerations, and person with acute disorders and higher education levels. Grootendorst [20] uses individual data from the 1990 Ontario Health Survey on the self-reported use of prescription drugs to compare alternative econometric models (Poisson model, negative binomial model, two part models) to study the different determinants of drug use. Chang/Trivedi [22] develop a theoretical model of self-medication behavior of a utility-maximizing consumer. Empirically, they especially focus on the role of income and health insurance on self-medication. Chan/Trivedi use individual cross-sectional data from the World Bank’s Living Standards Measurement Survey of Vietnam 1997–1998 and apply an econometric framework similar to [20]. They find that self-medication is an inferior good at high income levels and a normal good at low income levels. Insurance coverage strongly reduces self-medication. Costa-Font et al. [19] study the determinants of drug consumption in Catalonia using official survey data on an individual basis. Econometrically, a two-part model is used. They find evidence that gender, health status and the existence of insurance are significant predictors for out-of-pocket pharmaceutical expenditures. Income and cost sharing are strongly associated with drug use but not drug expenditures. McLeod et al. [24] analyze the financial burden of out-of-pocket expenditures for prescription drugs based on cross-sectional national survey data from Canada. They focus on the household drug budget share and estimate Engle curves for different drug budget shares using predicted values from the kernel conditional quantile estimator. Tavares [25] derives a formal model of self-medication based on the maximization of a utility function that depends on consumption and on health status. Thereby Tavares especially focuses on the role of time. Individual data from the Portuguese 4th National Health Survey (2005) are used in a probit model with self-medication as a binary variable. The results show significant effects for age and gender. As far as waiting time is concerned, Tavares offers mixed results.

Summarizing these studies we observe important differences in the study designs, the data used and the observed results. The majority of the studies are based on individual data; only [24] uses data from the household level. All studies using individual data except [21] directly control for the individual health status. Reports [22] and [25] base their empirical estimation on a theoretical model of drug consumption (self-medication), while the other papers use different steps of reduced forms. Reports [20–22] use two-part models in the econometric specification. Thereby, [20] and to some extent [22] present a detailed comparison and discussion of alternative model specifications. The clear focus of [19, 21] is price effects on the pharmaceutical consumption measured by the level of insurance coverage.

Our article contributes to the empirical research on OOPPE in several ways. First, it adds evidence from the perspective of the household and supplements the findings available from the individual level in the previous literature. Second, we use data from a health-care system that is based on Bismarckian principles and that holds a specific two-tiered institutional architecture of service provision and financing. Third, we keep in mind households’ decision-making process of either seeking professional health care or using self-medication, which leads to three types of out-of-pocket expenditures. We account for this fact by separating the available database into subgroups to analyze the different types of OOPPE separately. Finally, our source of information is the general household budget survey, while previous studies build on health-related surveys. Since national household budget surveys follow internationally agreed principals, our study also allows conclusions on whether budget surveys are an appropriate database to analyze the determinants of out-of-pocket health-care expenditures.

The remainder of the article is organized as follows. In “The policy setting of pharmaceutical consumption in Austria,” we present a brief overview of the main institutional characteristics of consuming pharmaceuticals in Austria. In “Databases and empirical approach,” we inform about the databases and derive conclusions for the empirical approach applied in the article. In “Econometric results and discussion,” we present the empirical results and discuss them. In “Conclusions,” we conclude our article.

## The policy setting of pharmaceutical consumption in Austria

In Austria authorities of the central state regulate basic dimensions of pharmaceutical consumption. They decide on the general preconditions and modes of market entry of pharmaceuticals, specifically on the separation between pharmaceuticals with obligatory prescription (8.026 pharmaceuticals in 2012; [26]) and over-the-counter (OTC) products (1.931 pharmaceuticals in 2012; [26]) and on pharmaceutical pricing. Thereby, the regulation of prices primarily focuses on maximum price margins of the wholesale firms and pharmacies, while factory prices are not regulated in Austria at this stage [27]. But this general regulation of market entry and prices primarily influences the provision of pharmaceuticals paid over the counter. There exists a second stage of public regulation of market entry and pricing conducted by the social health insurance system. Since social health insurance in Austria covers around 99.3 % of the whole population—excluding only marginal groups from public health insurance—this regulation has far-reaching consequences for pharmaceutical pricing and consumption [28].^1^ Only pharmaceuticals included in the positive list of the Reimbursement Code are paid by the social health insurance system. Thereby, the Reimbursement Code includes pharmaceuticals with and without obligatory prescription [29].^2^

Pharmaceuticals that are part of inpatient treatments are free for patients with social health insurance coverage. Their costs are included in the DRG-based hospital remuneration system [29, 30]. Pharmaceuticals that are part of outpatient treatments provided by GPs/specialists having a contract with the social health insurance system are basically free if they are included in the positive list of the Reimbursement Code. Patients have to pay a prescription fee for every pharmaceutical prescribed. This prescription fee is an absolute amount of money (in the years of the household survey: 2009: €4.90, 2010: €5.00) with no link to the price of the pharmaceutical. If the price of the pharmaceutical is below the prescription fee, patients only have to pay the price of the pharmaceutical. Calculated over the total range of pharmaceutical consumption financed by the social health insurance system, the prescription fee leads to a cost sharing of approximately 13 % [31]. If patients consume medical services supplied by private doctors, pharmaceuticals are paid by the social insurance system on request.

As far as the prescription fees are concerned, two schemes influence the financial burden of individuals (households). An exemption exists from the prescription fee and a prescription fee cap. An exemption is granted without application (1) for retired persons who draw a small pension from a public pension plan, (2) for persons with notifiable communicable diseases, (3) for members of the alternative civilian service including their relatives and (4) for asylum seekers. On application, an exemption from the prescription fee is granted for insurance members (including co-insured household members) with a household net income below the threshold value of the basic income maintenance system. Since 2008 the exemption from the prescription fee is accompanied by a prescription fee cap at a 2 % share of the annual net income.

Roughly 35 %^3^ of the population has signed contracts with private sickness funds, which predominantly offer additional coverage to services of the social health insurance system and/or improve the possibility to choose from a broader portfolio of providers/services within the system. But private health insurance does not play a significant role in financing pharmaceutical consumption. Only 0.2 % of the prescribed drugs and 1.7 % of the OTC products were paid by the private health insurance system in 2012 [33].

**Fig. 1:**
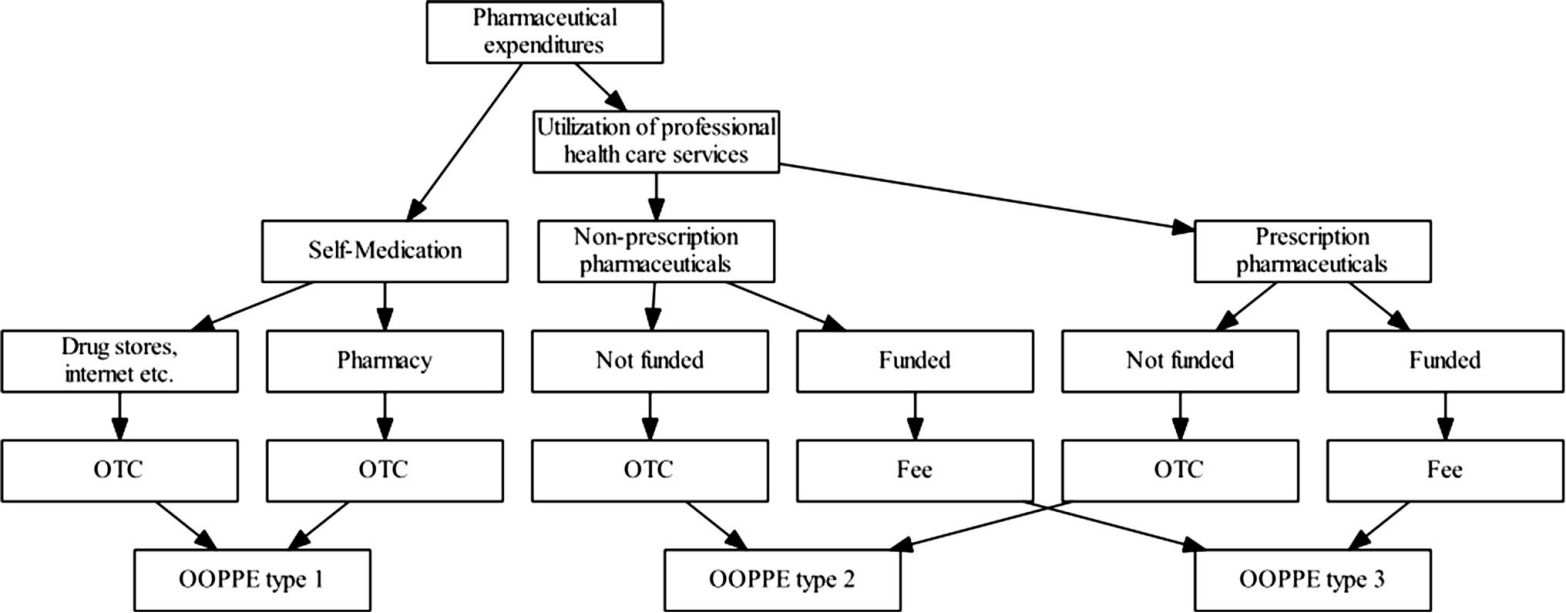
Utilization decision and types of OOPPE

Having in mind the institutional setting of consuming pharmaceuticals in Austria, we are able to identify possible treatment paths in the health care sector that might lead to OOPPE (see Fig. 1). In the first step the patient has to decide whether to rely on self-medication or to seek professional health care [22, 25]. In Austria, self-medication accounts for approximately 20 % of total pharmaceutical consumption (outside the hospital) and is mainly financed out of pocket [27]. If the patient decides to use outpatient medical services, pharmaceuticals with and without obligatory prescription are consumed. If they are funded by the social health insurance system, the patient only has to pay the prescription fee. If they are not funded, the patient has to pay the price. On average, 80 % of the pharmaceutical consumption (outside the hospital) in Austria is based on a prescription, 88 % of this consumption is refinanced by the social health insurance system, 11.8 % is paid out of pocket, and 0.2 % is refinanced by private sickness funds [33]. Summarizing, we end up with three forms of OOPPE (see Fig. 1): (1) OOPPE as a consequence of self-medication (OOPPE type 1); (2) OOPPE as a consequence of consulting the professional outpatient health-care sector and consuming pharmaceuticals that are not included in the Reimbursement Code of the social health insurance system (OOPPE type 2); (3) prescription fees for pharmaceuticals prescribed by the outpatient health care sector and paid by the social health insurance system (OOPPE type 3).

## Databases and empirical approach

### Databases

To analyze the socioeconomic determinants of OOPPE empirically, we use data from the household budget survey 2009/10 conducted by Statistics Austria. This periodically repeated survey is used to study the level and structure of private consumption of households within the System of National Accounts. The observation unit is the private household without institutionalized households. The total sample offered by Statistics Austria consists of 6534 households with 15,540 members. We exclude 747 households with unclear household and/or social health insurance status and use a final sample size of 5787 households.^4^

Information on the consumption behavior is gathered in two ways: (1) the diary approach and (2) the recall approach. Households participating in the survey are asked to fill in a diary over 14 days. The system results in 52 overlapping weeks of bookkeeping. The recall approach is used for consumer durables and irregular/seasonal expenditures within the last 12 months. Selected socioeconomic characteristics of the household are gathered by face-to-face interviews. All expenditures are recalculated into monthly expenditures. Following the expenditure classification of the household budget survey, pharmaceutical expenditures are included in the expenditure category “pharmaceuticals and medical products.” We only focus on pharmaceuticals here, thereby excluding dietary supplements. The subcategory “pharmaceuticals” is only separated into pharmaceuticals paid over the counter (OOPPE type 1 and 2) and prescription fees (OOPPE type 3). This inseparability of OOPPE of type 1 and 2 is an obvious backlash of our data set since self-medication—resulting in OOPPE of type 1 (Fig. 1)—is expected to be influenced by different covariates compared to the consumption of pharmaceuticals, resulting in OOPPE of type 2 (for economic models of self-medication, see [22, 23, 25]). The aggregation of the monetary consequences of the two different treatment paths blasts information. From the system of health accounts, we are able to conclude, that on average more than 80 % of the pharmaceutical expenditures paid over the counter result from the treatment path self-medication (OOPPE of type 1), but there is no guarantee that this share is unaffected by household characteristics [33].

### Empirical approach

For econometric and economic reasons, hurdle models, specifically two-part models, serve as methodological cornerstones to explain health-care utilization/expenditures [8, 13, 21]. The first part is a binary model that focuses on the separation between use (rs) and nonuse (rs). The second part explains the level/frequency of medical care use conditional on some use. Statistically, the split in the estimation procedure is substantiated by three specific characteristics of health-care utilization/expenditures and their consequences for the efficiency of estimation: (1) skewness, (2) excess zeros and (3) heavy right tails. From an economic perspective, the split in the estimation procedure is motivated by the fact that the two decision stages are characterized by differences of the involved decision makers. Thereby, the empirical strategy in the first step is in general based on structural or reduced-form equations of the Grossman model of demand for health services [34, 35]. The patient seeking care decides autonomously whether to seek professional diagnostic and curative medical help at all. The modeling of the second step is guided by principle-agent considerations leading to joint decisions of patients and their health-care suppliers.

In a nutshell, the ideal starting point of two-part models is the episode of medical treatment defined as a set of medical services received continuously by a patient in response to particular requests caused by a specific illness (for an extended discussion, see [15]). Thereby, the first step pictures a patient’s contact with medical providers, called the illness spell. The second step includes the result of the joint decisions captured by indicators such as health expenditures, treatment visits, referrals and prescriptions. It is obvious that the standards of data collection that enable us to differentiate between these two steps are challenging and hardly ever fulfilled by routine data. The previous literature is only partially aware of this fact in the choice of the empirical strategy [13, 20]. Only Santos Silvia and Windmeijer give a profound discussion of this problem and offer solutions for count data (physician visits) if the mix of the initial treatment spell and the following visits is not identifiable in the data set [14]. The description of the data processing for OOPPE in Austria makes clear that our data set does not perfectly fulfill the ideal preconditions for using a two-part model for several reasons. Basically, we have pharmaceutical expenditure data of a household gathered in a short observation period of 2 weeks. This observation period coincides with the length of an illness episode only by chance. The episode might start before the observation period and/or last longer and might lead to left and/or right truncation as a consequence. There is no possibility to separate between the initial spell and the following treatment contacts. The only information available is expenditure levels in a time period without knowing the number of contacts. These identification problems are multiplied by the fact that we observe OOPPE on the household level only. The same level of OOPPE is compatible with different utilization patterns of the single household members. Finally, the decision process leading to OOPPE and specifically the interaction of the two decision steps differ between the different types of OOPPE.

Taking into account these characteristics of our data set, the structural appeal of the two-part model is less obvious. We react to this fact and use different econometric approaches. In the case of OTC pharmaceuticals (OOPPE type 1 and 2), we apply a two-part model (TPM) and a one-stage generalized linear model (GLM). Considering the TPM, the first stage of the model predicts the likelihood of any OOPPE and was specified as Logit. The second part predicts the level of spending, conditional on having non-zero OOPPE. As an alternative modeling strategy, we use a GLM that estimates the parameters of the two processes jointly. To specify the GLM models. we proceed in the following way: We test for the kurtosis of the log-transformed OOPPE to determine the link function. Following the literature, the relationship between the variance and the mean is estimated by a modified Park test [36]. In this procedure, the squared residuals from a provisional log-transformed OLS model or a provisional GLM model are regressed on the predictions from the same model. The corresponding coefficient suggests either a constant variance model (*λ* = 0), a model whose variance is proportional to the mean (*λ* = 1), or the standard deviation proportional to the mean model (*λ* = 2). However, the best model specification falls typically between the two latter models. The performance of the chosen model will be evaluated by computing the mean absolute error, mean squared error and *R* ^2^ scores as suggested by Matsaganis et al. [11]. For both econometric approaches, we further use Pregibon’s Link test, Ramsey’s Reset test, a modified Hosmer-Lemeshow test, Cook’s distance and an overall goodness of fit test for the combined model to evaluate the fit of the chosen model.

In the case of prescription fees (OOPPE type 3), we recalculated the non-zero expenditures into the number of prescriptions by the application of prescription fee intervals. So our variable “prescription fees” pictures at once the household expenditures for prescription fees and the consumption of publicly financed pharmaceuticals. To deal with the distribution of the data, the high frequency and the expected heterogeneity (the different sources) of the zeros, we test several regression models: Poisson, a negative binomial model (NB), a zero-inflated negative binomial model (ZINB) and a hurdle model (two-part model for count data) (for a detailed discussion, see [20]). The goodness of fit of the corresponding models was evaluated by using the likelihood-ratio test to compare Poisson vs. NB and the (ZIP) vs. (ZINB). We further used the BIC and AIC statistics (Poisson vs. NB/ZIP/ZINB, NB vs. ZIP/ZINB and ZIP/ZINB) and the Vuong test (Poisson vs. ZIP, NB vs. ZINB, ZIP vs. ZINB) as well as the mean absolute error and mean squared error as model selection criteria as recommended in the literature [20, 37]. In contrast, in the hurdle model it is assumed that all zeros are from one source and that the non-zero part of the data follows a truncated Poisson or a truncated negative binomial distribution [37]. The model comparison of this positive part is undertaken by the likelihood ratio test, while the latter goodness of fit test encompasses Pregibon’s Link test and Ramsey’s Reset test.

No explicit behavioral model of OOPPE is put forward; in fact, a reduced form model is estimated. We extensively test for the household structure, which captures not only the size and composition of the household, but to some extent also pictures different phases in the lifecycle of a household (single, unmarried couple, married couple, full nest I, full nest II, empty nest). We further control for adults’ age, adults’ education level, household income, gender of the householders, the existence of early retirement individuals in the household and the socioeconomic surrounding of the household expressed by the degree of urbanization. In addition, we also test whether the type of public health insurance and the existence of private health insurances influence the OOPPE. Finally, we control for doctoral visits by any household member indicated by the out-of-pocket expenditures for physician services in the observation period and defined as dummy variables. Hereby, doctoral visits can be considered as a proxy for the low health status of at least one household member. We expect a positive effect on OOPPE, because physician contacts could be an indicator for a low health status and therefore might increase the demand for pharmaceuticals. Table 4 (in the “Appendix”) contains the detailed description of the variables employed in the study.

## Econometric results and discussion

Table 1 shows the descriptive statistics of the explanatory variables. Out of 5787 households, 1150 have non-zero expenditures for prescription fees with a mean per month of 34.47. In the case of OTC pharmaceuticals, the non-zero mean expenditures of the 1559 households sum up to 41.00. In the raw data, we observe substantial differences of OOPPE levels depending on the household structure, adults’ age, adults’ education level and the type of public health insurance. The differences are more pronounced for OTC pharmaceuticals compared to prescription fees.

**Table 1:** Descriptive statistics of the variables employed according to both types of OOPPE

Total households	Prescription fees				OTC pharmaceuticals			
	Average exp.		Exp. >0		Average exp.		Exp. >0	
	Mean	SD	Mean	SD	Mean	SD	Mean	SD
Household structure								
Single person I	3.39	12.76	30.30	25.43	6.86	26.12	41.87	52.10
Single person II	5.97	15.72	30.69	22.65	7.53	25.49	35.52	45.60
Unmarried couple	4.20	15.45	33.52	30.63	13.66	31.10	48.32	41.88
Married couple	8.07	23.84	37.82	39.33	9.99	25.56	38.27	37.77
Empty nest	4.14	11.83	24.51	18.18	14.69	36.72	42.24	52.15
Full nest I	5.65	18.49	27.48	32.67	13.63	34.79	42.49	50.51
Full nest II	17.67	32.44	47.80	37.53	13.27	35.53	42.76	53.05
Married couple w/o childs	6.58	15.42	28.54	20.18	12.84	34.25	41.65	51.15
Single parents	2.41	9.08	24.12	17.52	9.57	24.68	37.38	36.70
Degree of urbanization								
High urbanization	6.69	20.82	36.01	35.77	11.55	31.35	42.48	47.99
Average urbanization	7.43	20.88	35.71	32.96	11.13	28.00	38.72	40.76
Low urbanization	6.61	17.63	32.32	26.25	10.52	32.34	41.29	53.27
Adults’ age structure								
Age <25	1.31	6.15	21.05	14.25	6.11	21.80	43.84	42.44
Age 25–45	3.10	10.55	23.74	19.05	11.98	32.86	43.32	50.49
Age 45–65	7.37	20.88	34.15	33.27	10.29	28.10	38.50	43.26
Age 65–85	14.09	28.31	43.87	34.49	11.68	33.66	41.16	52.77
Adults’ education level								
Primary education	7.64	19.71	37.79	28.03	5.91	18.66	34.05	32.43
Other education	7.04	19.86	34.21	31.43	11.49	32.01	41.09	49.50
Tertiary education	4.04	17.57	30.79	39.38	14.55	34.20	45.90	47.52
Insurance characteristics								
GKK	6.66	19.21	34.04	30.90	10.05	29.15	39.08	46.61
BVA	7.61	20.13	34.73	30.154	15.17	37.27	45.59	52.85
SVA	6.76	22.94	37.16	42.18	12.01	32.67	45.77	50.34
SVB	6.82	17.31	36.02	23.18	6.62	18.28	34.96	28.06
Private health insurance^a^	8.30	23.98	36.68	38.82	13.38	33.29	42.21	47.80
Private health insurance^b^	7.62	20.83	35.61	32.15	12.62	35.40	42.53	54.36
Total households	6.85	19.67	34.47	31.54	11.05	30.91	41.00	48.16
*N* (households)	5787		1150		5787		1559	

^a^Corresponds to one adult of the household who has an additional private health insurance

^b^Corresponds to both adults of the households that have an additional private health insurance. This also includes households consisting of one individual (single person I and single person II). Dummy variables for female householders and income are not reported in the table. For definitions of the particular variables employed, see Table 4 in the “Appendix”

Table 2 shows the econometric results of the TPM and GLM for OTC pharmaceuticals (OOPPE type 1 and 2). The probability for OTC spending is strongly influenced by the life cycle of the household. The signs of the coefficients are highly plausible; the size of the coefficients are partially unexpected. There is some evidence that the probability of OTC spending is lower in regions with a low degree of urbanization. As far as self-medication is concerned, the difference in the relative time costs of using professional health services compared to pharmacies could be an important covariate to explain this fact [22], but our data do not allow to test for this hypothesis. The positive relationship of the OTC spending with age—especially in the older age groups 45–65 and >65—is expected and well documented in previous empirical research. The education level increases the probability for OTC spending significantly. The health insurance characteristics of the household—either private or public—are of very limited influence on probability of OTC spending. This follows our expectations for several reasons. The general preconditions of consuming pharmaceuticals (e.g., pharmaceuticals included in the positive list, level of the prescription fee, exemptions from the prescription fee) do not differ among the different public sickness funds (GKK, BVA, SVA, SVB) compared in the sample. Differences might be caused only indirectly by differences in the socioeconomic characteristics of the different groups of publicly insured (e.g., opportunity costs of time when being ill, schedules of physician services remuneration). As already mentioned, private health insurance only plays a very limited role in financing pharmaceutical consumption in Austria. So we expect only indirect effects on the OOPPE-levels caused by, e.g., higher risk aversion of individuals with private health insurance or effects of the remuneration system of private health insurance on treatment behavior of health-care providers. Household income and female gender of the householder increases the probability of positive OTC spending. Finally, we observe that doctoral visits of a household member in the same period increase the possibility of OTC expenditures. According to the Box-Cox test (*λ* near zero), we use for the second stage an OLS model with a log-transformed dependent variable denoted as log OLS in Table 2. In contrast to the highly significant covariates of the first stage, the covariates of the second stage remain largely insignificant. The income elasticity of OTC expenditures is near zero (0.073), but the coefficient is insignificant. One interpretation of the results on the second stage could be that the probability of OTC consumption of a household systematically depends on several household characteristics, while the level of expenditures is highly stochastic in the short time perspective represented in our data.

**Table 2:** Econometric results of the two-part model and GLM for OTC pharmaceuticals

	Two-part model				GLM^b^	
	Logit		Conditional (log OLS)^a^			
	Coeff.	Rob. SD	Coeff.	Rob. SD	Coeff.	Rob. SD
Household structure						
Single person II	−0.055	0.165	−0.046	0.112	−0.059	0.171
Unmarried couple	0.636***	0.196	0.201	0.130	0.741***	0.194
Married couple	0.365**	0.180	0.007	0.121	0.393**	0.196
Empty nest	1.094***	0.171	−0.003	0.115	0.425**	0.196
Full nest I	0.790***	0.170	0.027	0.114	0.845***	0.175
Full nest II	0.417**	0.191	0.061	0.120	0.737***	0.188
Married couple w/o childs	0.762***	0.205	0.010	0.137	0.652***	0.211
Single parents	0.373**	0.177	−0.029	0.120	0.302	0.209
Degree of urbanization						
Average urbanization	0.088	0.092	−0.065	0.060	−0.054	0.090
Low urbanization	−0.159*	0.087	−0.062	0.057	−0.170*	0.093
Adults’ age structure						
Age 25–45	0.337	0.256	−0.088	0.177	0.413	0.256
Age 45–65	0.555**	0.262	−0.196	0.182	0.467*	0.267
Age 65–85	0.935***	0.283	−0.148	0.191	0.817***	0.276
Adults’ education level						
Other education	0.473***	0.137	0.022	0.091	0.503***	0.143
Tertiary education	0.697***	0.186	0.123	0.119	0.702***	0.185
Insurance characteristics						
BVA	0.181**	0.091	0.094	0.059	0.206**	0.087
SVA	−0.087	0.132	0.147*	0.087	0.129	0.160
SVB	−0.257	0.238	0.097	0.167	−0.179	0.245
Private health insurance^c^	−0.029	0.109	−0.042	0.072	−0.023	0.112
Private health insurance^d^	0.074	0.089	0.008	0.059	0.044	0.103
Other characteristics						
Early retired	−0.142	0.158	0.012	0.107	−0.326**	0.156
Female householder	0.264***	0.092	0.004	0.063	0.195*	0.103
Doctoral visits	0.367***	0.108	0.061	0.068	0.346***	0.010
Income (log)	0.188**	0.090	0.073	0.062	0.138	0.092
Constant	−4.035***	0.704	2.780***	0.486	−0.258	0.720
Observations (households)	5787		1559		5775	

^a^Log-transformed dependent variable

^b^GLM with log-link and Poisson distribution

^c^Corresponds to one adult of the household who has an additional private health insurance

^d^All adults of the household have an additional health insurance. This also includes households consisting of one individual (single person I and single person II). Reference groups: single person I, high urbanization, age class 18 - 25, primary education, GKK, no additional private health insurance, male householder, not early retired and no doctoral visit

Significance level: *** p < 0.01, ** p < 0.05, * *p* < 0.1

Columns 5 and 6 of Table 2 show the results of the GLM. We tested for the kurtosis of the log-transformed OOPPE that takes the value 2.99, which is very close to 3 and therefore justifies a log link function. As mentioned in the empirical approach, we performed a modified Park test. The corresponding estimates are *λ* = 1.55 (provisional OLS model with log-transformed dependent variable) and *λ* = 1.20 (provisional GLM model) favoring a variance proportional to the mean model. In the evaluation of the model performance, the variance proportional to the mean model clearly outperforms the alternative.^5^ We used this specification in our estimation. Our results reveal a significant effect of the household structure, adults’ age, adults’ education level and doctoral visits. The same is true with restrictions for the degree of urbanization. Income remains insignificant, which is also true for private and public insurance characteristics (exemption: BVA). Considering both model specifications for the analysis of OTC pharmaceuticals, the one-stage GLM predominately approves the findings of the TPM except for household income and the status of early retirement.

Table 3 shows the econometric results for the second form of OOPPE—the prescription fees (OOPPE type 3). We compared the performance of different econometric models using likelihood ratio tests, BIC, AIC and Vuong tests as model selection criteria. The zero inflated negative binomial model (ZINB) fits better than all other models. This is also true for the NB in the positive part of the hurdle model. Therefore, Table 3 only presents the results for the hurdle model—with the NB specification in the first step—and the ZINB model when focusing on the characteristics of the zeros.

**Table 3:** Econometric results of the hurdle model and zero-inflated negative binomial regression model for prescription fees

	Hurdle model				Zero-inflated negative binomial			
	Logit		Negative binomial		Logit		Negative binomial	
	Coeff.	Rob. SD	Coeff.	Rob. SD	Coeff.	Rob. SD	Coeff.	Rob. SD
Household structure								
Single person II	−0.062	0.190	−0.023	0.141	0.072	0.211	−0.010	0.137
Unmarried couple	−0.154	0.245	0.392*	0.217	0.302	0.273	0.393*	0.215
Married couple	0.368*	0.203	0.489***	0.177	−0.217	0.227	0.501***	0.176
Empty nest	0.651***	0.199	0.577***	0.156	−0.504**	0.222	0.597***	0.155
Full nest I	0.632***	0.204	0.235	0.190	−0.581**	0.250	0.242	0.200
Full nest II	0.472**	0.196	0.154	0.174	−0.439*	0.232	0.172	0.180
Married couple w/o childs	0.336	0.234	0.071	0.178	−0.333	0.268	0.079	0.176
Single parents	−0.459*	0.243	0.027	0.202	0.498*	0.278	0.055	0.204
Degree of urbanization								
Average urbanization	−0.012	0.103	−0.019	0.082	0.000	0.115	−0.030	0.082
Low urbanization	−0.133	0.096	−0.131	0.081	0.083	0.107	−0.145*	0.083
Adults’ age structure								
Age 25–45	0.599	0.375	0.434	0.304	−0.426	0.468	0.431	0.294
Age 45–65	1.283***	0.378	0.804**	0.318	−1.011**	0.474	0.793**	0.312
Age 65–85	1.850***	0.392	0.948***	0.325	−1.564***	0.487	0.939***	0.319
Adults’ education level								
Other education	0.136	0.134	-0.066	0.090	-0.163	0.144	−0.067	0.089
Tertiary education	−0.260	0.218	0.127	0.179	0.327	0.234	0.134	0.181
Insurance characteristics								
BVA	−0.094	0.109	−0.017	0.082	0.095	0.120	−0.010	0.082
SVA	−0.264*	0.154	−0.041	0.136	0.278	0.173	−0.021	0.135
SVB	−0.263	0.253	0.066	0.166	0.308	0.270	0.101	0.163
Private health insurance^a^	0.087	0.125	−0.047	0.108	−0.120	0.145	−0.053	0.110
Private health insurance^b^	0.061	0.101	0.067	0.078	−0.048	0.114	0.060	0.080
Other characteristics								
Early retired	−0.313*	0.181	0.157	0.149	0.370*	0.190	0.159	0.148
Female householder	0.183*	0.107	−0.051	0.096	−0.212*	0.120	−0.048	0.090
Doctoral visits	0.446***	0.121	−0.160*	0.093	−0.558***	0.144	−0.173*	0.095
Income (log)	0.099	0.108	−0.146*	0.080	−0.161	0.121	−0.154*	0.082
Constant	−3.734***	0.873	1.303**	0.648	3.725***	0.984	1.366**	0.643
Observations (households)	5787		1150				5787	
Lnα							−0.938***	0.141
α							0.392	0.055

^a^Corresponds to one adult of the household who has an additional private health insurance

^b^All adults of the household have an additional private health insurance. This also includes households consisting of one individual (single person I and single person II). Reference groups: single person I, high urbanization, age class 18–25, primary education, GKK, no additional private health insurance, male householder, not early retired and no doctoral visit

Significance level: *** p < 0.01, ** p < 0.05, * *p* < 0.1

The results of the hurdle model are shown in the left part of Table 3. The first part of the model is defined as logit and demonstrates the importance of the households’ life cycle. Especially households consisting of more household members (married couples, empty nest, full nest I and full nest II) increase the probability of having prescriptions significantly. Surprisingly, single parents have a significantly lower probability of non-zero prescriptions. Adults’ increased age (age groups 45–65 and 65–85), female householders and doctoral visits within the observation period increase the probability, while early retired householders and households who are insured at SVA decrease it significantly. The existence of private health insurance, income, education and the degree of urbanization have no effect on the probability of prescriptions. The second part of the model is defined as zero-truncated Poisson regression. Concerning the household structure, the results show that the log count of prescriptions increases significantly for unmarried and married couples, empty nests and adults with increased age (age groups 45–65 and 65–85) and decreases with income and doctoral visits of the affected household members.

The right part of Table 3 shows the results of the ZINB regression model. The splitting function (logit) reveals the covariates that influence the probabilities of true zeros. As expected, the coefficients of the covariates show a similar size but reversed signs compared with the first step of the hurdle model: e.g., the probability of a true zero in the prescription variable strongly decreases with age. Additionally, the existence of doctoral visits, female householders and children (full nest I, full nest II) decreases the probability of true zeros. In contrast, single parents and householders who are early retired increase the log odds of true zeros. The level of prescriptions (NB) sharply increases with age, is inverse to the degree of urbanization and decreases with income. The expected number of prescriptions for unmarried couples is 1.48 times the expected number of prescriptions for a single person I while holding all other variables constant. Furthermore, married couples, empty nests and households without doctoral visits in the observed time period have a higher expected number of prescriptions than the particular reference groups (see column 5 and 6 of Table 3). Public insurance characteristics and the existence of private health insurance remain insignificant in both estimation stages. The same is true for the level of education. Using AIC, we also tested the hurdle model and ZINB model [37] and found a better model fit for the ZINB model.

Our study differs from the previous research in several respects. We use data from the household level, while the majority of the previous studies use individual data. Our study analyzes two different forms of OPPE, while the previous research concentrates on either self-medication or prescriptive drugs. Econometrically, our study is most similar to the studies of [20, 22]. The results of our study are to some extent in line with the previous literature [19, 20, 23–25]. We confirm the effect of age on OTC pharmaceuticals and prescription drugs and the mixed results for income. In line with [23, 25], we find a positive effect of education on the consumption of OTC pharmaceuticals. In contrast to previous findings, we do not find an effect of private health insurance on the probability and level of OOPPE. The same is true for the type of public insurance. This is an indication that public coverage against the risk of pharmaceutical expenditures in Austria is high and homogenous. In line with the majority of the previous research—an exemption is [21]—we do not test for the price elasticity of pharmaceutical consumption directly, but control for the existence of different forms of insurance coverage.

We conclude our discussion section by pointing to possible limitations of our study. In our presentation of the empirical design of the study, we already pointed to several challenges. In line with [19–21, 23, 24], we do not apply a theoretical model of pharmaceutical consumption, but test reduced forms. This means that our study to some extent has an explanatory character. Our data picture the level of households. This allows us to test for household characteristics, e.g., the household structure. But we should be aware of the fact that conclusions from the household level to the individual level should be drawn with caution. Although the decision to consume pharmaceuticals might be influenced by household characteristics, it remains an individual decision and mainly depends on individual characteristics, which are masked to some extent on the household level. This has important consequences for the reproduction of the decision-making process when consuming pharmaceuticals. Additionally, the covariates age and education have a different meaning on the household level compared to the individual level, which might lead to differences in the estimated effects. Finally, we want to discuss the suitability of data from general consumption surveys to picture the consumption of health care goods and to identify the covariates driving the consumption level and structure. General consumption surveys are well-established instruments of economic accounting and follow internationally agreed standards of reporting. This in principle qualifies them as an information source also for the consumption of health care goods and services. For several reasons, general consumption surveys offer high data quality concerning consumption expenditures and selected socioeconomic characteristics of the households. On the other hand, they only include rudimental information on socioeconomic characteristics of individuals (households) that are able to explain the utilization of health care services, e.g., the consumption of pharmaceuticals. Such characteristics are indicators for the health status, the need of long-term care and the individual disease profile over a longer time period. The empirical literature clearly reveals that the health status—e.g., measured by health expenditure in the past—is an important predictor of health-care expenditures and explains most of the variance in regression models. The missing of such information is an important source of unobserved heterogeneity and is also an indication for the low explanatory power of the used covariates in our estimations. The explanatory power of the covariates used in the previous literature, which controls for the health status—see especially [19, 20, 22, 25], is substantially higher compared to our results. In addition, general consumption surveys normally do not include information on the supply characteristics of health-care services (e.g., distribution of pharmacies and physicians, provision/utilization of public health care services), which might influence the utilization decision heavily. Finally, the information from consumption surveys is period based and does not allow a separation in different steps of the utilization process. Overall, we conclude that the information on health expenditures (e.g., pharmaceutical expenditures) from consumption surveys primarily should be used to study the covariates of pharmaceutical consumption/expenditure from a distributive point of view. They allow clear insights into the role of the household structure (life cycle of the household), the income and education in pharmaceutical consumption.

## Conclusions

This article analyzes the socioeconomic determinants of OOPPE of private households in Austria using data from the household budget survey 2009/2010. Empirically, the data show substantial differences in the expenditures between households in different stages of their lifecycle. The characteristics of the data set (information from the household level, specification of the dependent variable, period based instead of illness episode based data, short observation period) pose several challenges to the choice of the estimation strategy. The advantages of the widely used TPM are no longer obvious. We react to this fact and compare and use different econometric approaches (TPM, GLM, Hurdle model, ZINB). Overall, we find that several household characteristics—especially the household structure, adults’ age, income, doctoral visits and adults’ education level—have strong effects on the probability and level of OOPPE. This is especially true for OTC pharmaceuticals, but to a reduced degree also for prescription fees. On the other hand, we do not find substantial effects of the type of public health insurance and the existence of private health insurance. The results of our study complete the picture of the covariates of OOPPE on the individual with evidence from the perspective of the household. The present study can help health policy decision makers to identify inequalities in pharmaceutical consumption and obtain insights into the covariates causing them.

## Appendix

See Table 4.

**Table 4:** Overview of the used variable specification and the corresponding percentage of observations

Explanatory variables	Percentage of observations	SD	Definition
Household structure			
Single person I	12.98	0.44	Household consists of one adult, single
Single person II	16.80	0.49	Household consists of one adult, either married, divorced or widowed
Unmarried couple	5.93	0.31	Household consists of two adults, unmarried
Married couple	10.13	0.40	Household consists of two adults, married, members are below 60 years
Empty nest	13.98	0.46	Household consists of two adults, married, members are above 60 years
Full nest I	12.37	0.43	Household consists of two adults, members are below 40 years, at least one child
Full nest II	13.36	0.45	Household consists of two adults, members are above 40 years, at least one child
Married couple w/o childs	6.22	0.32	Household consists of more than three adults, married, no children
Single parents	7.97	0.36	Household consists of one adult, at least one child
Degree of urbanization			
High urbanization	35.65	0.63	Areas with a population of at least 50,000 and more than 500 inhabitants per square kilometer
Average urbanization	25.90	0.58	Areas with a population of at least 50,000 and 100–500 inhabitants per square kilometer
Low urbanization	38.45	0.64	All other areas
Adults’ age structure			
Age <25	3.59	0.24	Average age of both adults. Refers to householder, if household consists of one adult
Age 25–45	37.67	0.64	Average age of both adults. Refers to householder, if household consists of one adult
Age 45–65	39.31	0.64	Average age of both adults. Refers to householder, if household consists of one adult
Age 65–85	19.42	0.52	Average age of both adults. Refers to householder, if household consists of one adult
Adults’ education level			
Primary education	12.74	0.44	Both adults have a primary education level. This also includes households consisting of one adult
Other education	78.43	0.54	Both adults have a mixed or secondary education level. This also includes households consisting of one adult
Tertiary education	8.83	0.37	Both adults have a secondary education level. This also includes households consisting of one adult
Insurance characteristics			
GKK	70.43	0.60	Workers in the private sector. Refers to householder’s insurance type
BVA	18.28	0.51	Public servants. Refers to householder’s insurance type
SVA	8.36	0.36	Employers. Refers to householder’s insurance type
SVB	2.92	0.22	Farmers. Refers to householder’s insurance type
Additional private health insurance (1)	11.61	0.42	One adult of the household has an additional health insurance
Additional private health insurance (2)	20.67	0.53	All adults have an additional health insurance. This includes households consisting of one adult
Other characteristics			
Early retired	5.98	0.31	Householder is retired and below 60 years
Female householder	32.73	0.62	Householder is female
Doctoral visits	10.23	0.40	At least one household member had a doctoral visit
Income	2986.4	2025.56	Monthly household income in euros

## Footnotes

1 Thereby, the public health insurance consists of different sickness funds. They are separated by territorial and occupational characteristics and have restricted autonomy in defining the terms of consumption of publicly paid health-care services. Workers in the private sector (76 % of the population) are covered by nine sickness funds operating at the level of the nine provinces (named GKK in our tables later on). Farmers (4 %; sickness fund named SVB), employers (8 %; sickness fund named SVA) and public workers (8 %; sickness fund named BVA) are covered by nationwide operating insurance institutions.

2 In 2009 the total expenditures for pharmaceuticals without obligatory prescription amounted to 526 million euros [12 % of the total pharmaceutical market (hospitals excluded)]. Eight percent of the expenditures for these products is refunded by the social health insurance system [27].

3 Information from 2012; for details, see Versicherungsverband Österreich [32].

4 No direct information on the public health insurance status of household members is provided in the household survey. We derive the insurance status from occupational characteristics of the household members. This might lead to minor blurring.

5 According to the results of Cook’s distance, we excluded 12 observations and based our findings of the GLM on 5775 households.
